# Supplementary material for: The Role of Medicago lupulina Interaction with Rhizophagus irregularis in the Determination of Root Metabolome at Early Stages of AM Symbiosis
Source: Plants (Basel). 2022 Sep 7;11(18):2338. doi: 10.3390/plants11182338 (PMC9501341; doi:10.3390/plants11182338)
Supplement: Supplementary file 1 [file plants-11-02338-s001.zip › Supplementary Figure S1-S3.pdf]

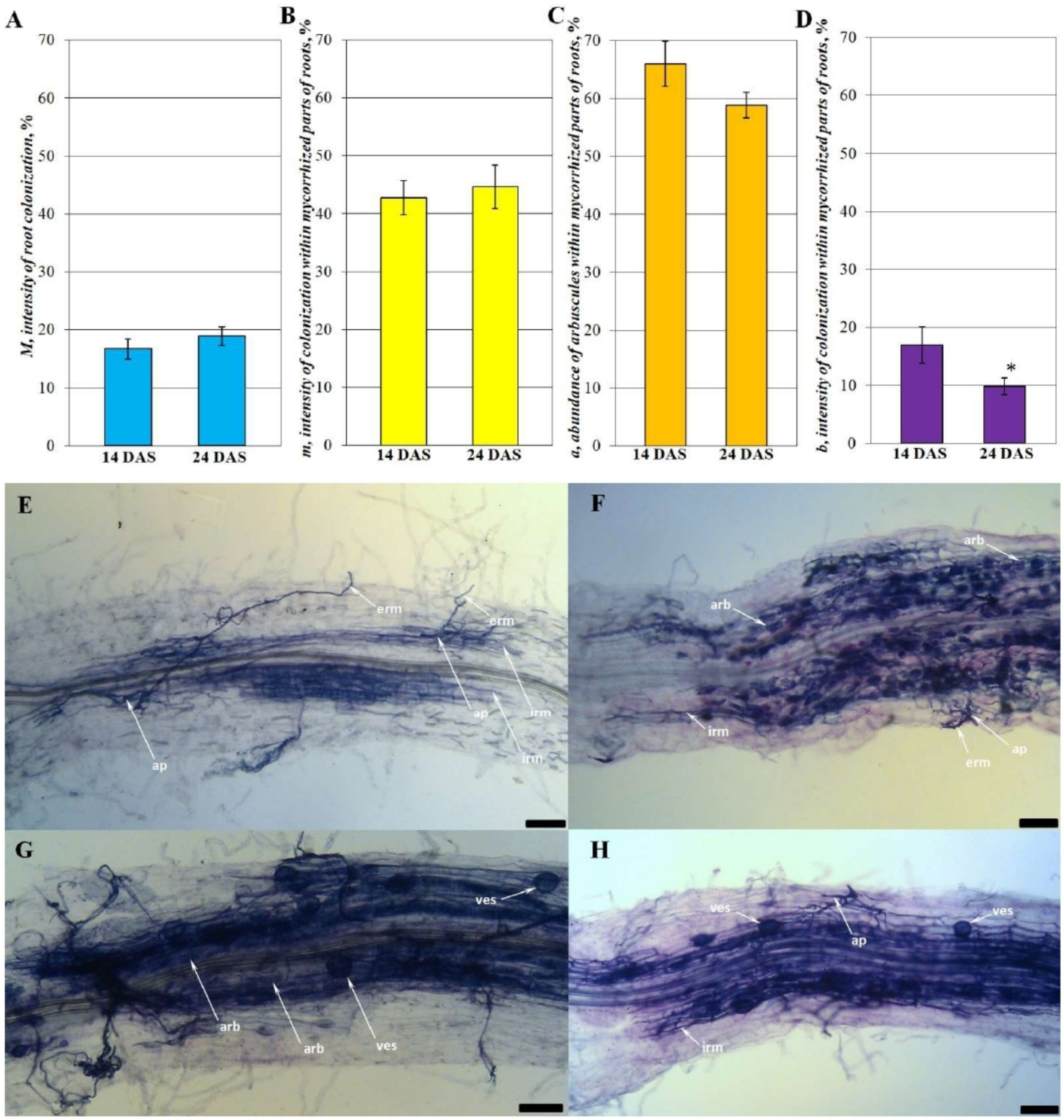

Figure S1. Mycorrhization parameters for *M. lupulina* roots infected by *R. irregularis*: **A.** *M* – the intensity of root cortex colonization; **B.** *m* – the intensity of colonization within individual mycorrhizal roots; **C.** *a* – the abundance of arbuscules in mycorrhized parts of roots; **D.** *b* – the abundance of vesicles in mycorrhized parts of roots; **E.** Appressoria and AM at 14 DAS; **F.** Arbuscules in AM at 24 DAS; **G.** Arbuscules and vesicules in AM at 14 DAS; **H.** Vesicules in AM at 24 DAS. "DAS" is the day after sowing and inoculation, "erm" – extraradical micelium,"irm" – intraradical micelium, "ap" – appressorium, "arb" – arbuscule, "ves" – vesicle. The asterisk indicates significant ($P<0.05$) difference in *b*. The scale bar is 100 μm.

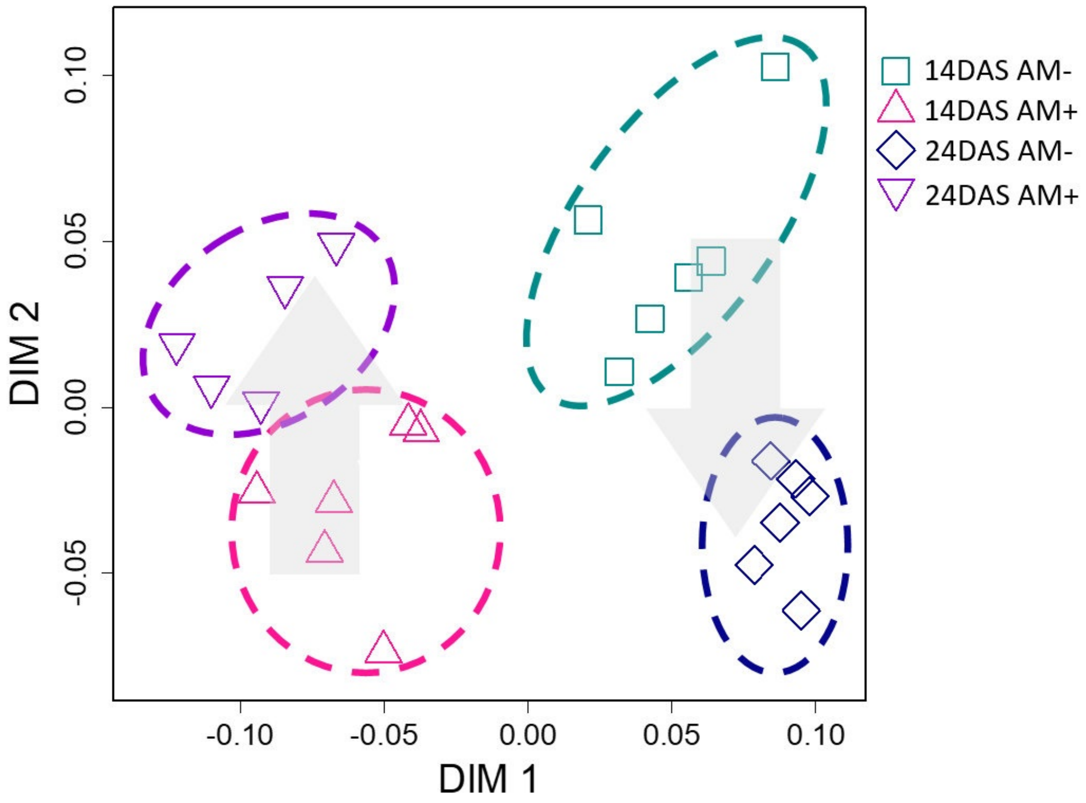

Figure S2. Representation metabolite profiles in low-dimensional spaces revealed from MDS, using 1-*r* as a measure of distance, where *r* is Pearson's correlation coefficient.

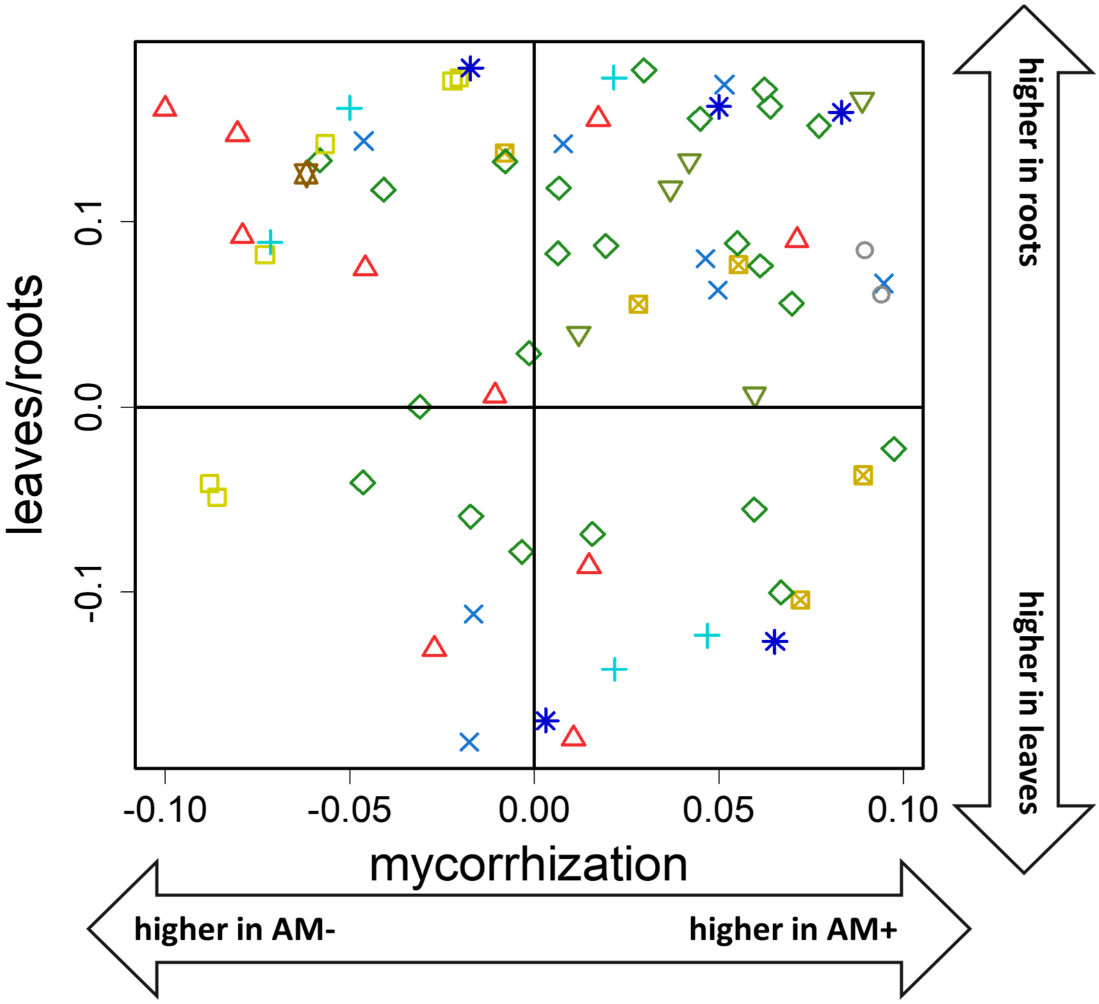

Figure S3. Comparison of mycorrhization effects and organ specificity. SUS plot in the space of the loadings from two OPLS-DA models for discrimination of mycorrhized (AM+) and nonmycorrhized (AM-) roots (abscissa) and roots/leaves (ordinate) at both time points. Symbols are the same as in Figure 5.

The following supporting information can be downloaded at: www.mdpi.com/xxx/s1, **Table S1**: Peak areas normalized per internal standard.
